# Gut Microbiome as a Potential Factor for Modulating Resistance to Cancer Immunotherapy

**DOI:** 10.3389/fimmu.2019.02989

**Published:** 2020-01-17

**Authors:** Lin Shui, Xi Yang, Jian Li, Cheng Yi, Qin Sun, Hong Zhu

**Affiliations:** ^1^Department of Medical Oncology, Cancer Center, West China Hospital, Sichuan University, Chengdu, China; ^2^Department of Pharmacy, The Affiliated Traditional Chinese Medicine Hospital of Southwest Medical University, Luzhou, China; ^3^Drug Research Center, The Affiliated Traditional Chinese Medicine Hospital of Southwest Medical University, Luzhou, China

**Keywords:** gut microbiome, cancer immunotherapy, resistance, PD-1/PD-L1, CTLA-4

## Abstract

Gut microbiota refers to the diverse community of more than 100 trillion microorganisms residing in our intestines. It is now known that any shift in the composition of gut microbiota from that present during the healthy state in an individual is associated with predisposition to multiple pathological conditions, such as diabetes, autoimmunity, and even cancer. Currently, therapies targeting programmed cell death protein 1/programmed cell death 1 ligand 1 or cytotoxic T-lymphocyte antigen-4 are the focus of cancer immunotherapy and are widely applied in clinical treatment of various tumors. Owing to relatively low overall response rate, however, it has been an ongoing research endeavor to identify the mechanisms or factors for improving the therapeutic efficacy of these immunotherapies. Other than causing mutations that affect gene expression, some gut bacteria may also activate or repress the host's response to immune checkpoint inhibitors. In this review, we have described recent advancements made in understanding the regulatory relationship between gut microbiome and cancer immunotherapy. We have also summarized the potential molecular mechanisms behind this interaction, which can serve as a basis for utilizing different kinds of gut bacteria as promising tools for reversing immunotherapy resistance in cancer.

## Introduction

More than 1,000 species of microorganisms inhabit the human intestines (1). Different types of microorganisms exist in different proportions in the intestines. They not only restrict but also depend on each other for growth and survival, and form an ecological balance of quality and quantity. Generally, there are three categories of gut bacteria based on their functions in the host: (1) symbionts, with mutual benefit to the host, account for nearly 90%; (2) conditioned pathobionts, normally harmless but produce disease due to fortuitous events that affect the host; and (3) pathobionts, disease-causing organisms (2–4). Gut microbiota is initially acquired from mothers during delivery and lactation and is followed by stable colonization of the host until 3 years of age. Subsequently, the microbial composition is shaped by external environmental factors (5–7). In other words, both host genetics and environmental factors are significant in determining the composition of intestinal microbial communities.

Various kinds of gut microbial commensal maintain intestine homeostasis by processing complex dietary ingredients (such as fiber) into digestible metabolites and facilitate immune surveillance to resist invading pathogens (8). Nevertheless, equilibrium between gut microbiota and host is disturbed by the action of antibiotics, exposure to pathogens, altered dietary patterns, and other stimuli, such as changes in weather or disruption of the circadian clock (9). This is known as dysbiosis, which is defined as a change in the diversity, composition, and structure of intestinal flora. Dysbiosis is associated with several physiological as well as pathological changes in the host. For example, gut dysbiosis induced by an antibiotic treatment causes overgrowth of commensal fungus, increases plasma concentration of prostaglandin E2, and promotes M2-macrophage differentiation, all of which leads to heightened airway allergy and inflammation (10).

In recent years, scientists have discovered that the gut microbiota of humans and other animals play a much bigger role in maintaining host health than previously thought. Petersen and his colleagues found that *Clostridium* prevents weight gain by blocking the ability of the gut to absorb fat. In the experiment, *Clostridium* was the only bacteria in the intestinal tract of mice, and the *Clostridium-*harboring mice were leaner and less fat than the germ-free (GF) mice (11). In other words, gut bacteria could control host body weight in that study. In addition, gut microbiota play a role in maintaining appropriate amount of skeletal muscles and their function in mice (12). Furthermore, Bradley et al. discovered that gut microbes increase interferon α/β signals of host lung stromal cells, thereby improving the ability of these cells to resist influenza virus infection (13). Various studies now suggest that dysbiosis gives rise to metabolic disorders (obesity, diabetes, etc.) (14–16), autoimmunity (systemic lupus erythematosus) (17, 18), inflammatory bowel disease (IBD) (19), or central nervous system disorders (multiple sclerosis) (20, 21). Moreover, research has found an unexpected link between cancer and gut microbiota. With all of this scientific evidence, it can be hypothesized that dysbiosis can also affect the process of oncogenesis, tumor progression, and response to cancer therapy (22). Riquelme et al. also found that fecal microbiota transplant (FMT) from long-term survivors boosted the immune response and limited tumor growth in mouse models by altering the tumor microbiota (23). Such results present a promising opportunity to improve the cancer treatment by changing the tumor immune microenvironment through regulation of gut microbiota.

Tumor immunotherapy has become a hot topic for cancer treatment. Present tumor immune-targeted treatments are focused on toll-like receptor (TLR) agonists, vaccines, immune checkpoint inhibitors (ICIs), and adoptive T-cell therapy (ACT). ICIs play a pivotal role in the treatment of various advanced-stage cancers, as evidenced by monoclonal antibodies blocking the programmed cell death protein 1/programmed cell death 1 ligand 1 (PD-1/PD-L1) and the cytotoxic T-lymphocyte antigen-4 (CTLA-4) (24). However, only less than half the patients respond to ICIs initially, and another subgroup becomes resistant during continued treatment, suggesting the presence of natural-acquired or therapy-induced resistance (25). Because of the life-threatening side effects or decrease in the quality of life, it is important to accurately identify the subset of patients who benefit most from ICIs. PD-L1 overexpression (26) and gene analysis (mutational landscape or mismatch repair deficiency) (27) have proven to be instrumental in predicting potential clinical outcomes of PD-1/PD-L1 blockade therapy. Snyder et al. (28) have performed whole-exome sequencing on patients with melanoma treated with CTLA-4 blockade and showed that cancer genomics can help in determining patient response to immunotherapy. Over recent years, a growing number of studies have also emphasized that the gut microbiome could modulate response to cancer immunotherapy. Some of these studies, however, were conducted using innovative treatments without extensive application in patients and, therefore, require further work to unlock the mystery of microbial modulation of various anticancer immunotherapies.

## The Relationship Between Gut Microbiota And The Intestinal Mucosal Immune System

The mucosal immune system is an independent immune system with a unique structure and functions. The mucus layer of the intestine is composed of mucus proteins secreted by goblet cells, mucins (rich in antimicrobial peptides) secreted by intestinal epithelial cells, and secretory immunoglobulin A produced by B cells. It can effectively inhibit the adhesion and colonization of bacteria in intestinal epithelium, a physical barrier mediated through tight junctions that helps in separating bacteria and harmful antigens from the internal environment. Mucosal immune system is also comprised of gut-associated lymphoid tissue that mainly includes histological lymphoid tissue and diffused lymphocytes. The former includes Peyer's patches, isolated lymphoid follicles, and mesenteric lymphoid nodes. The latter mainly refers to lymphocytes scattered in lamina propria and epithelial cell layer, such as dendritic cells (DCs), T cells, and B cells.

The relationship between gut microbes and the host immune system is predicted to be more unique in the gut than on other internal microbial environments. It is widely considered that commensal bacteria can induce a protective immune response to ensure host–microbial mutualism. Moreover, a direct role of intestinal bacteria in the development and maturation of immune system has been reported. The function of gut bacteria in shaping host immunity also appears in studies investigating how the gut microbiome could indirectly modulate immune response via metabolism (29). Studies on GF mice have shown reduced intestinal bacterial diversity resulting in development of chronic immune disease. Furthermore, switching of the microbiota into the inflammation-inducing type has also been observed in many chronic inflammatory diseases (30). In general, gut microbiota can affect the function of intestinal mucosal immunity. Therefore, the interaction between gut microbiota and intestinal immune system is deemed essential for maintaining mucosal homeostasis.

Intestinal microbiota can enhance IgA production in the intestinal tract by regulating the response of B cells. It has been found that compared with SPF mice, GF mice have a dysplastic mucosal immune system and poorly developed Peyer's patches, with only few germinal centers and significantly decreased number of IgA and IgA^+^ plasma cells. However, the production of IgA can be restored after colonization of gut bacteria in GF mice (31). This suggests that intestinal bacteria play an important role in the differentiation of IgA^+^ plasma cells and the production of IgA in the intestines. IgA is mainly produced and secreted by IgA^+^ plasma cells in the lamina propria of intestinal mucosa. After binding to the polyimmunoglobulin receptor produced by intestinal epithelial cells, IgA is transported and released into the intestinal cavity to bind with gut microorganisms or dietary components (32). The adhesion and wrapping function of IgA can avoid direct contact between harmful antigens and intestinal epithelium and maintain the integrity of intestinal barrier. At the same time, IgA also regulates the composition of gut microbiota (33). Angiopoietin-4 is bactericidal protein, which can be secreted into the intestinal cavity to fight against microorganisms. The results from reverse transcription quantitative PCR in a study have shown that, in GF mice, the expression level of angiopoietin-4 was significantly lower than that in conventional mice (34). Therefore, it is reasonable to consider that intestinal microbiota is indispensable for mucosal immunity.

Gut microbiota can also maintain the balance between inflammatory response and immune tolerance by regulating T cell differentiation. Naive CD4^+^ T cells can proliferate and differentiate into various subtypes, including T-helper cell 1 (Th 1), Th2, Th17, and regulatory T cells (Treg) cells. Among them, Th17 cells are mainly proinflammatory and are positively associated with autoimmune diseases. Treg cells as well as Th17 cells in the lamina propria are capable of controlling the inflammatory response and maintenance of immune tolerance. In recent years, a large number of studies have shown that the main mechanism by which the gut microbiota maintains the balance between inflammatory response and immune tolerance is through moderating the differentiation of Th17 cells and Treg cells. Corroborating this, Ivanov et al. found that the number of Th17 cells in the small intestine of GF mice was lower than SPF mice but increased significantly after cofeeding with SPF mice. It was also reported that the number of Treg cells in the lamina propria of the colon of GF mice was lower than that of SPF mice (35). A study by Britton et al. indicated that compared to microbiota from healthy donors, transfer of IBD microbiota into GF mice increased the numbers of intestinal Th17 cells and Th2 cells but decreased the numbers of RORɤt Treg cells (36). All of the above-mentioned evidence support that the adhesion of intestinal bacteria to intestinal epithelial cells may regulate the generation and differentiation of Th17 cells by sending a certain signal, but the exact mechanism remains to be further explored.

Epithelial ɤδ T cell subgroup is part of a larger group of residing lymphocytes known as intraepithelial lymphocytes (IEL). Although the microbiota has an immense effect on the composition and number of αβ T cells, the development and numbers of ɤδ IELs show no difference between GF and WT mice. Nonetheless, microbiota do restrict the function of gut ɤδ IEL. Transplantation of microbiota harvested from conventional mice into GF mice can induce production of several antimicrobial peptides, including regenerating islet-derived protein 3 gamma (RegIIIɤ) (37), a C-type lectin that recognizes and binds to Gram-positive bacteria and forms a hexameric membrane-penetrating pore leading to direct bacterial killing (38). In short, these studies illustrate that detection of invasive bacteria by ɤδ IELs balances intestinal microbiota through crosstalk with adjacent epithelial cells, thereby acting as a vital component of the immune system.

## Regulating Effect Of Systemic Immune System Exerted By Gut Microbiome

Intestinal flora is not only related to intestinal immune diseases but also has an important impact beyond the gut. The famous “microflora hypothesis” points out that other than specific limited infections, an overly clean western lifestyle limits universal microbial contact and, thus, changes the colonization of the intestinal microbiota of infants along with undermining the development of the immune system and triggering various immune diseases. For instance, symptoms of gut microbiota disorder usually appear within a course of 6 months in early arthritis patients (39). Intestinal flora is considered to affect systemic immune diseases through the following ways: (1) The small molecular substances generated by intestinal flora enter the blood circulation and affect the immune response of distal organs. A study showed that the microbial homeostasis was disturbed after treatment with antibiotics, as a result of which a specific fungus proliferated excessively. This was followed by increased concentration of prostaglandins in plasma that boosted the polarization of M2 macrophages in the lung and caused allergic inflammation (40). (2) Through the common mucosal immune system existing throughout the whole body. An emerging perspective described the mucosal immune system as a complete network of tissues that protects the host, prevents infection, and resists environmental interference. In this system, immune activation of one local site may result in altered immune microenvironment at another distal site. Consequently, it is easy to understand why exposure to mold spores or ovalbumin in the nose is more likely to cause allergic respiratory diseases under circumstances where the microbial homeostasis in the intestines is broken (41). (3) The signals produced by gut microbiota or recognized by TLR have an effect on extraintestinal diseases due to alteration of the immune response. (4) Gut microbiota can also manage the development of systemic immune cells. Hence, it seems that the more beneficial bacteria in the gut, the more complete the immune system, and the more conducive it is toward adapting to the external environment.

## Research Concerning The Modulatory Effect Of Gut Microbiome On Cancer Immunotherapies

**Figure 1:**
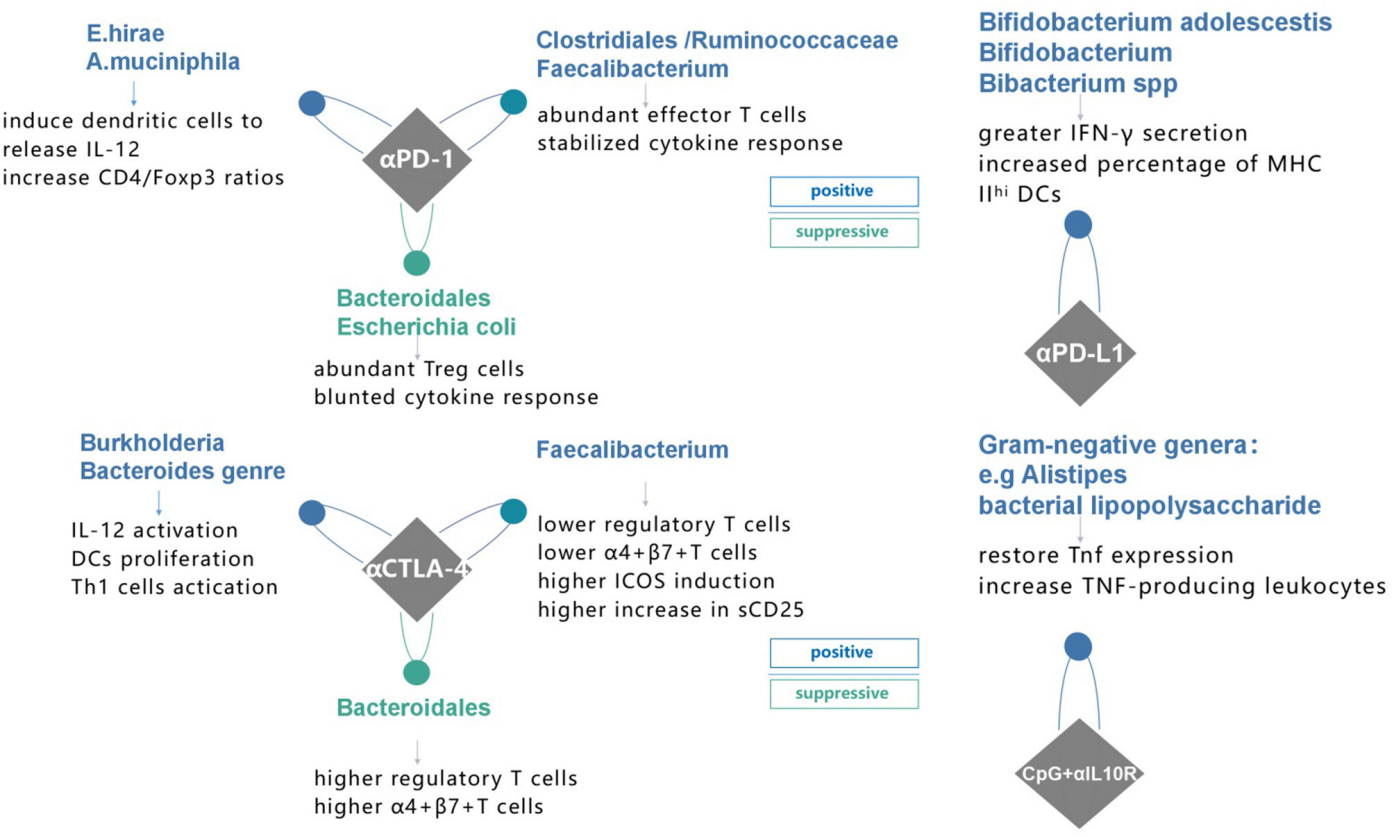
Specific species of bacteria have proven to affect immune response to four different immunotherapies and possible mechanisms in recent studies. PD-1 programmed death receptor-1, PD-L1 programmed death-ligand 1, CTLA-4 cytotoxic T lymphocyte-associated protein 4, αPD-1, anti-PD-1 therapy; αPD-L1, anti-PD-L1 therapy; αCTLA-4, anti-CTLA-4 therapy; CpG^+^αIL10R, TLR9 ligand CpG plus anti-IL10R antibody.

Earlier, multiple studies showed a correlation between gut microbiome and cancer therapy, such as the chemotherapy-elicited anticancer immune response of cyclophosphamide (42, 43). It was postulated that microbiota changes the tumor microenvironment (TME) to improve immunomodulatory effects. However, these results raise new questions, such as whether a causal link with gut microbiome and cancer immunotherapy exists or not. Here, we reviewed the data from recent advances to address such questions (Figure 1).

### Immune Checkpoint Inhibitors

PD-1 and its ligands (PD-L1/PD-L2), along with CTLA-4 and its ligand B7, are a part of CD28 and B7 families that have a crucial role in T-cell exhaustion (44). ICIs aim to counteract T-cell inhibition to resurrect the antitumor activity of the immune system.

PD-1 is a major inhibitory receptor expressed on tumor-infiltrating lymphocytes (TILs) as well as activated natural killer T cells (45), B cells (46), and DCs (47). However, its ligands (PD-L1/PD-L2) are expressed constitutively on the membrane of antigen-presenting cells (APCs). Binding reaction on regulatory T cells (Tregs) triggers the conversion of naive CD4^+^ T cells to Tregs (48), resulting in improved inhibitory activity and maintenance of Foxp3 expression through blockade of AKT-mammalian target of rapamycin signaling and increased phosphatase and tensin homolog deleted on chromosome 10 activity (49). Therefore, PD-1/PD-L1 pathway not only represses the activation and proliferation of T effector cells to escape from the immune surveillance and promote tumor growth and metastasis (50) but also increases the function of immunosuppressive Tregs (51). PD-1 inhibitors reactivate the function of T cells to restore antitumor immunity with considerably less toxicity (52). Over the past few years, a mounting number of PD-1/PD-L1 targeted monoclonal antibodies have been recommended by clinical guidelines and widely used in various solid or hematological tumors (53).

In 2015, Sivan et al. (54) proposed that *Bifidobacterium*, a specific taxon of microbial commensals, strengthened antitumor immunity and raised the efficacy of PD-L1 blocking therapy. First, they observed that two genetically similar C57BL/6 mice implanted with subcutaneous B16.SIY melanoma from two different facilities [Jackson Laboratory (JAX) and Taconic Farms (TAC)] exhibited distinct differences in tumor growth rate, TILs response, and CD8^+^ T cell numbers. However, these differences were eliminated after cohousing, which meant that commensal microbiota might mediate an anticancer effect. To directly verify the hypothesis, researchers transferred JAX or TAC fecal material from one mouse to another by oral gavage before tumor implantation. Compared to saline or TAC fecal material, prophylactic transfer of JAX fecal suspensions to TAC-recipient mice impeded tumor growth and enhanced infiltration of tumor-specific CD8^+^ T cells. Furthermore, it showed that the combination of JAX fecal transplantation with PD-L1 blockade enhanced tumor control, suggesting that microbiota can alter not only the antitumor immunity but also the response to PD-L1 inhibitors. Comparative analysis of the fecal bacterial content between JAX and TAC mice was done by sequencing 16S ribosomal RNA. JAX mice were shown to have 257 more taxa than TAC mice, and it was the *Bifidobacterium* operational taxonomic unit_681370 that increased over 400 times in JAX-fed TAC mice (54).

Since the above experiments were on preclinical tumor mice models, clinical experiments to identify the specific bacteria genres playing a decisive role in human immunity were still needed. In the following years, researchers continued to carry out an analogous analysis of the human microbiome (55). They collected stool materials from 42 patients with metastatic melanoma before administering PD-1 blockade, and the clinical response rate was 38%, showing that there were 16 responders (R) and 26 non-responders (NR). The authors selected 10 bacterial species that were different between R and NR, 8 of those were found to be more abundant in R (including *Bifidobacterium*, also observed in preclinical studies). Next, the authors transferred feces from three R and three NR patients into tumor-bearing GF mice. Two of the three human microbiota-colonized mice with the R stool sample had inferior tumor growth. As expected, PD-1 inhibitor exerted its antitumor activity in mice colonized with R bacteria but was ineffective in NR bacteria colonized mice, thus confirming *in vivo* the influence of gut microbiome on human immunity.

**Figure 2:**
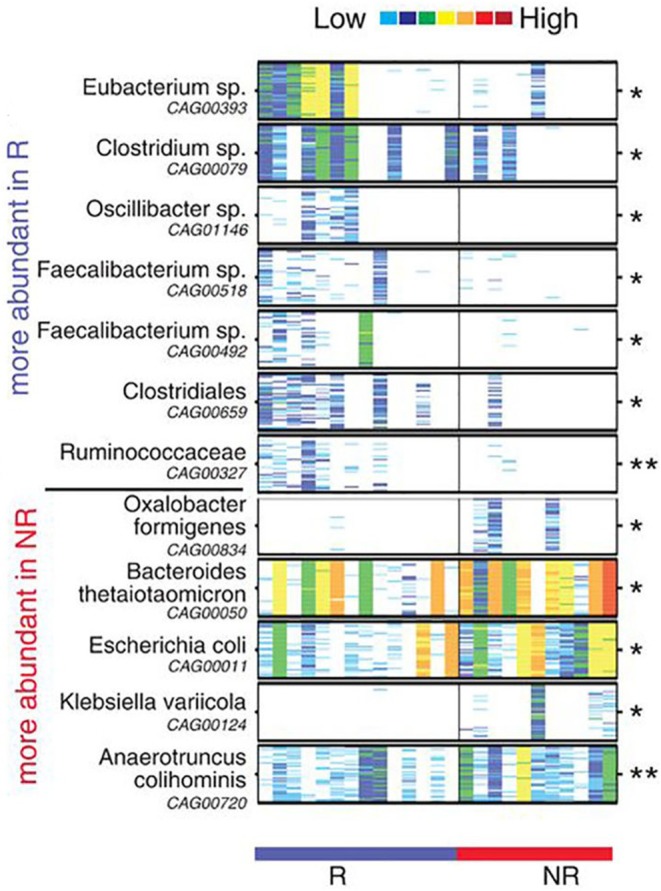
Compositional differences in the gut microbiome are associated with responses to anti-PD-1 immunotherapy. Pairwise comparisons by MW test of abundances of metagenomic species (MGS) identified by metagenomic WGS in fecal samples (*n* = 25): Responder (R) (*n* = 14, blue), Non-responder (NR) (*n* = 11, red). *p < 0.05, **p < 0.01. Colors reflect gene abundances visualized using “barcodes” with the following order of intensity: white (0) <light blue <blue <green <yellow <orange <red for increasing abundance and each color change corresponds to a 4x fold abundance change. In these barcodes, MGS appear as vertical lines (co-abundant genes in a sample) colored according to the gene abundance [The figure is reprinted with permission from Gopalakrishnan et al. (57)].

This conclusion was supported by other researchers. Routy et al. (56) explored the association of dysbiosis with epithelial tumors to understand whether simultaneous use of antibiotics (ATB) generates primary resistance to ICIs in mice and patients (56). Their results showed that the antitumor effect was compromised in ATB treatment group, with progression-free survival (PFS) and overall survival (OS) being significantly shorter compared to that of the control group, demonstrating that ATB could be used as a predictive marker for measuring ICIs resistance. Similarly, using the shotgun sequencing for quantitative metagenomics of the fecal sample, *Akkermansiacea muciniphila* and *Enterococcus hirae* were shown to be significantly abundant in patients with best clinical response to ICIs (PFS > 3 months). Moreover, Gopalakrishnan et al. (57) evaluated the oral and gut microbiome of 112 melanoma patients treated with PD-1 blockade via 16S sequencing and discovered a higher multiplicity of bacteria in patients with prolonged PFS. They showed an abundance of *Clostridiales/Ruminococcaceae/Faecalibacterium* in R and *Bacteroidales/Escherichia coli* in NR (Figure 2) (57). Patients with more *Faecalibacterium* also had a significantly prolonged PFS with higher level of effector T cells and a stabilized cytokine response to PD-1 blockade. In contrast, patients with more NR microorganisms often had a shortened PFS and a higher level of Tregs with a blunted cytokine response. To further confirm the cause and effect relationship between microbiota and PD-1 blockade efficacy, two research groups performed FMT from R and NR cancer patients to recolonize GF mice or the ATB-treated SPF mice, followed by inoculation with tumor cells and treatment with PD-1 blockade. Compared to NR-FMT mice, transplantation of fecal microbiota from R patients delayed tumor growth, increased the accumulation of CXCR3CD4^+^ T cells and CD8^+^ T cells, and upregulated PD-L1 expression in the TME, thereby counteracting the impaired anticancer ability against PD-L1 blockade. Similar effects were not observed although in mice treated with NR patient bacteria (56, 57). Furthermore, FMT from NR patients to GF mice led to the development of resistance for PD-1 blockade, with colonization of *A. muciniphila* and *E. hirae* being able to reverse the compromised efficacy (56).

Jin et al. (58) developed a clinical analysis to explore the relationship between gut microbiome and therapeutic outcomes in Chinese patients with advanced non-small-cell lung carcinoma who were being treated with PD-1 blockade therapies. According to their results, responding patients harbored higher diversity and stable composition of innate gut microbiome during treatment and also had significantly prolonged PFS. In detail, *Alistipes putredinis, Bifidobacterium longum*, and *Prevotella copri* were enriched in responders, whereas *Ruminococcus_unclassified* was found mainly in non-responding patients. As expected, a greater frequency of memory CD8^+^ T cell and natural killer cell subgroups was also observed in the periphery blood of responding patients.

Other than the microbiota colonized in the intestines, microbiota that migrates into the peritumoral immune microenvironment can also influence response to cancer immune therapies. Pushalkar et al. (59) showed a difference in bacterial composition between normal pancreas and pancreatic ductal adenocarcinoma (PDAC). Bacterial ablation led to a reduction in myeloid-derived suppressor cells, increased M1 macrophage differentiation, enhanced CD4^+^ T-cell differentiation and CD8^+^ T-cell activation. In addition, PD-1 expression upregulation caused by bacterial elimination could reverse immune tolerance to benefit the PD-1 blocking therapy.

Another predominant inhibitory regulator of T-cell activation is ipilimumab (60), an antibody that blocks CTLA-4, approved in 2011 by the Food and Drug Administration for significantly raising survival benefits in metastatic melanoma patients (61). CTLA-4 and CD28 are present on the surface of T cells and compete to unite with B7 costimulatory molecules on APCs (62). When CTLA-4 transmits an inhibitory signal to T cells, full activation of CD4^+^ or CD8^+^ T cells is interrupted, thus impeding the adaptive immune response against tumors (63). This is how ipilimumab can block the interaction between CTLA-4 and B7 to revive the antitumor response of T cells (64). This means that CTLA-4 not only acts as a competitive antagonist for activating T cell but also impairs immune response by mediating T-cell signaling pathways (65, 66). Research by Vétizou et al. (67) showed that efficacy of CTLA-4 blockade partly depends on the host microbiota. In this study, CTLA-4 blocking therapy was found to be effective against sarcoma, melanoma, and colon cancer in SPF mice but not in GF mice. A combination of ATBs (ampicillin, colistin, and streptomycin) or imipenem alone destroyed the function of CTLA-4 inhibitors against the tumor. These preliminary results showed that gut microbiota might be indispensable for the antitumor efficacy of anti-CTLA-4 treatment. In GF or ATB-treated mice, activation of effector CD4^+^ T cells and TILs elicited by CTLA-4 blockade was considerably dampened. Moreover, recolonization of ATB-treated or GF mice with commensal bacteria, such as *Bacteroides* and *Burkholderia* genres, could restore the CTLA-4 blockade-mediated anticancer responses. In fact, dietary supplements with *Bacteroides fragilis* induced Th1 immune responses in the tumor-draining lymph nodes prompted the maturation of DCs in the TME and facilitated the restoration of the clinical response to CTLA-4 blockade. Interestingly, it was discovered that CTLA-4 blocking therapy could reverse the gut bacteria repertoire, causing a decrease in beneficial bacteria and an increase in bacteria that blunt the response to ipilimumab. This observation offered new insights for acquired resistance during CTLA-4 blockade. In other words, management of gut microbiome elicits modification of TME and turns on a switch for the development of resistance to immune therapy, which enables tumor cells to escape immune surveillance.

**Figure 3:**
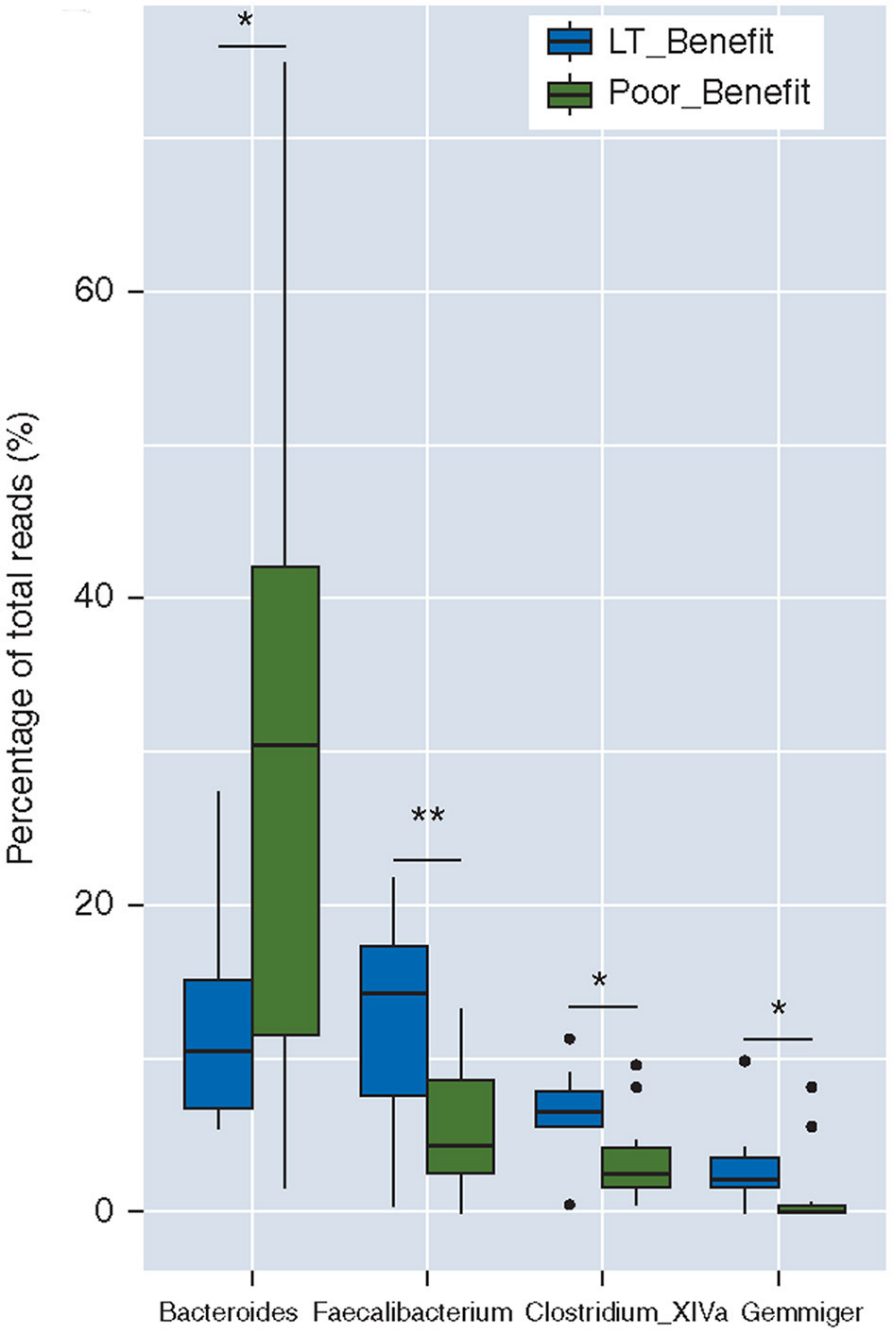
Baseline gut microbiota as a predictor of response to ipilimumab. Boxplot of the percentages of four dominant (>1% of total reads) genera differentially represented between both groups, i.e., *Bacteroides, Faecalibacterium, Clostridium XIVa*, and *Gemmiger*; LT_Benefit, long-term benefit vs. Poor Benefit; *p < 0.05; **p < 0.001 [The figure is reprinted with permission from Chaput et al. (68)].

However, discrepancies exist between such a suppressed effect of CTLA-4 blockade on gut microbiota. According to Chaput et al. (68), the dominant bacterial phyla remained stable during ipilimumab treatment course. Thus, whether ipilimumab could induce a gut microbial dysbiosis deserves reliable proof by further detailed studies. Chaput et al. also addressed the question of whether microbiota composition at baseline was associated with a subsequent clinical response. Twenty-six melanoma patients treated with ipilimumab were enrolled in this study, and analysis of fecal microbiota composition of patients were in accordance with an earlier study: *Bacteroides* were present in majority of the patients with poor clinical benefit, but a higher proportion of *Faecalibacterium* existed in patients with longer survival (68) (Figure 3), who also had a smaller proportion of baseline Tregs and a lower frequency of effector T cells compared to those with shorter PFS.

### CpG-Oligonucleotide Immunotherapy

Pattern recognition receptors (PRRs) are an important part of host innate immune system for sensing invading microorganisms (69). PRRs recognize pattern-associated molecular patterns (PAMPs) and initiate a cascade of intracellular signaling pathways resulting in the activation of immune cells (70). The most-studied PRRs are TLRs; they detect PAMPs from various pathogens (71). TLRs connect the innate and adaptive immune system by recognizing pathogens via DCs and in turn boosting T and B cell responses, thereby supporting the use of synthetic TLR agonists for the benefit of cancer vaccines (72).

Interleukin-10 (IL-10) is a pleiotropic cytokine generated by a wide variety of cells. IL-10 could downregulate the function of APCs and delay T-cell activation to inhibit the generation of proinflammatory cytokines. On the contrary, it can also suppress tumor progression by IL-10 signaling in CD8^+^ TILs cells. Hence, IL-10 plays a paradoxical role in cancer immunotherapy through activation or inhibition of IL-10 signaling (73). Therefore, it is important to select target cells based on treatment goals (74). IL-10 signaling blockades are still under exploration and are a long way from being used in cancer therapy due to unclear therapeutic efficacy and safety. An *ex vivo* study assessed the effect of selective blockade of IL-10R on CD4^+^ T cell response to hepatitis C virus antigens. The results showed that suppressing IL-10R could promote CD4^+^ T cell proliferation and increase hepatitis C virus-specific interferon-gamma (IFN-ɤ) production (75).

Intratumor injections of TLR9 ligand CpG and anti-IL10R antibody are able to reinforce antitumor efficacy in C26 and B16 animal models. The therapeutic effect resulting from enhanced tumor-infiltrating DCs and macrophage infiltrate switching from M2 to M1 consequently produce increased IL-12 and tumor necrosis factor (TNF) (76).

In a complementary study (77), tumor-bearing mice treated with an intratumor injection of CpG-oligodeoxynucleotides and anti-IL-10R were randomized into control and ABT-treated groups (vancomycin, imipenem, and neomycin). Based on the results, it was identified that ABT decreased the number of TNF-secreting cells and cytokines in major histocompatibility complex (MHC)-II monocytes, macrophages, and DCs (77). Gavage administration of bacterial lipopolysaccharide (LPS) considerably increased *Tnf* expression and TNF-producing cells in tumors of ABT-treated wild-type mice but not *Tlr4*^−/−^ mice (absence of the Tlr4 receptor for LPS) (77). The ability of ABT oral gavage to reduce gastrointestinal microbial load in the absence of bacterial DNA in tumors and the restorative effect of LPS suggested that gut microbiome could modulate the immune response in the TME. Taken together, the participation of commensal bacteria is found to be imperative for CpG/anti-IL10R immunotherapy (77).

### Adoptive Cell Therapy

Adoptive cell therapy (ACT) refers to the expansion and modification of cancer-cognate lymphocytes *in vitro* for infusion, with the goal of improving immune function and characteristics (78). TCR-engineered T cells or CAR-engineered T cells increase the efficacy of the antitumor immune response (79). Uribe-Herranz et al. (80) showed the correlation between the gut microbiome and the efficacy of ACT therapy. The researchers developed two mouse models (JAX/HAR) of cervical and lung cancer with E6/E7 human papillomavirus protein expression and different types of gut microbiome, and transferred CD3^+^ T cells, generated by vaccination of donor mice with human papillomavirus-associated cancers, to the lymph-depleted tumor-bearing animals. ACT was more effective in HAR mice receiving ACT and almost completely abrogated tumor growth. Examination of fecal bacteria revealed that HAR mice harbored a more diverse range of *Bacteroidetes* taxa than JAX mice (80). ACT efficacy increased when the bacterial composition was altered following depletion of Gram-positive taxa by antibiotics, but it was unchanged after depletion of Gram-negative bacteria. The ACT protocol was followed after FMT from JAX to HAR mice pretreated with antibiotics to compromise the resident gut microbiota. It was reasonable to hypothesize that the combination of vancomycin and ACT in HAR mice after FMT resulted in a similar response to ACT as in JAX mice. The above results showed that the gut microbiome plays an important role in the antitumor effectiveness of ACT.

## Correlation Of The Microbiome And Adverse Events Of Immunotherapy

Apart from the modulation of efficacy, the microbiome may also predict the susceptibility to immunotherapy-associated adverse events. The immune-mediated, new-onset colitis is the most frequently occurring adverse event during immunotherapy and is primarily controlled by dose decrement or application of corticosteroids. In a prospective study on immune-mediated colitis, bacteria of the *Bacteroidetes* phylum, which includes some of the most beneficial bacterial species, were enriched in colitis-resistant patients (81). Moreover, the absence of genetic pathways associated with polyamine transport and biosynthesis of vitamin B increased the susceptibility to colitis (81). The immunomodulatory molecule named polysaccharide A, generated by the human symbiont, can prevent or even cure colitis in animals (82). *Bifidobacterium* treatment reduces weight loss following CTLA-4 blocking therapy in a standard colitis mouse model (83). In addition, decreased secretion of inflammatory cytokines such as KC, IL-6, and CFS3 as observed with the same growth kinetics of the tumor, indicating that *Bifidobacterium* mitigates gut immunopathology without compromising on the effects of immunotherapy. Identification of microbial biomarkers may help in reducing the development of inflammatory complications caused by cancer immunotherapy.

Wang et al. (84) reported the first case of ICI-associated colitis successfully treated with FMT, with reconstitution of the gut microbiota and a relative increase in the proportion of Treg cells within the colonic mucosa. These preliminary data provided evidence that modulation of the gut microbiome may abrogate ICI-related colitis. However, a limitation of this study is the small number of samples that were used in the study. Therefore, additional studies are critically needed to assess the utility of this approach as well as to provide further mechanistic insights.

## Role Of Specific Species Of Intestinal Microorganisms Or The Microbial Biodiversity In The Immune Response

Genetic variation between different hosts is closely related to the architecture of individual gut microbiota. Classic twin studies, for example, have shown that the gut microbiota of identical twins is significantly more similar than that of fraternal twins (85). However, the main environmental factors, including diet lifestyle, evolutionary history, immune system, age, and antibiotics still influence the composition of the gut microbiota (86). The majority of studies have reported that there is a positive correlation between microbial diversity and the immune response. In Chinese patients with advanced non-small-cell lung carcinoma, the higher was the diversity of the gut microbiota, the better was the response observed to anti-PD-1 immunotherapy (58). However, some researchers believe that the gut microbiome could induce tumor progression. Sethi et al. (87) found that gut microbiota depletion in a mice model significantly increases IFN-ɤ-producing T cells and correspondingly decreases the IL 17A and IL 10-producing T cells, thereby reducing tumor growth. Uribe-Herranz et al. (80) have shown that, in a cervical cancer mice model treated with adoptive T cell therapy, the concurrent use of vancomycin can reduce tumor progression, mainly due to the opposite effect of the vancomycin-sensitive Gram-negative bacteria. These two contradictory results could not be easily reconciled based on our current knowledge on the topic.

As a microbial ecosystem, the composition and numbers of microbial community fluctuate in a dynamic equilibrium under the influence of external factors. At least for now, it is difficult to accurately identify the individual genres that specifically modulate the immune response among the thousands of bacteria populations. Moreover, it is likely that the effect of a single kind of microorganism is not enough to cause such a qualitative leap. Perhaps, the changing tendency of the microbiota architecture or dominant bacteria is the critical regulatory factor. Tanoue et al. (88) have isolated successfully IFNɤ^+^ CD8 T-cell-inducing bacteria strains from healthy human microbiota and indicated that they work as a consortium in which the four non-*Bacteroidales* species are effector elements and the seven *Bacteroidales* play a supporting role. Because of inductive accumulation of colonic IFNɤ^+^ CD8 T cell, colonization with the 11 strains exhibited enhanced clearance of tumor cells when combined with ICIs therapy.

## Mechanisms Involved In The Reversal Of Resistance To Immunity By The Gut Microbiome

Some of the above-mentioned studies showed that bacteria can differentially affect the T-cell immune response and potentially remold the “cold” or suppressive TME into a “hot” or responsive one. However, the possible mechanisms underlying this concept are still under investigation. To resolve this issue holistically, data collected from diverse microbial genera ought to be evaluated in consideration of the multidimensional view of tumors, taking into account the multiple influencing factors such as TME and the host characteristics involved in therapeutic efficacy (89). It is likely that metabolic changes induced by the microbiota could coordinate the TME, enabling the revival of T-cell functions to counteract tumor-induced immunotolerance (90).

### Increasing Production of Cytokines

Recent results have reported significantly higher levels of IFN-ɤ in the tumor-draining lymph node and spleen after introduction of beneficial bacteria (54). IFN-ɤ, an important cytokine involved in antitumor immunity, could upregulate MHC expression on tumor cells and M1 macrophages and facilitate the recognition and elimination of transformed cells (91, 92). IFN-ɤ is a dimer that combines with specific heterodimer receptors to activate JAK1/2 for subsequent regulation of the induction of STAT1 dimerization and genetic transcription (93). CTLA-4 blocking therapy of tumor-bearing mice also reportedly improves IFN-ɤ signaling in T cells (94). Loss of IFN-ɤ signaling could induce resistance to anti-CTLA-4 treatment (95). In line with these findings, microbes may reverse the resistance to immunotherapy through reactivation of IFN-ɤ signaling pathways.

In several prior investigations, it was shown unequivocally that oral supplementation with favorable microbiota after FMT from NR donors could rescue the efficacy of PD-1 blockade. Routy et al. (56) proposed that the restoration may be IL-12 dependent and increase the recruitment of CD4^+^ T lymphocytes into tumor beds. IL-12 is a pleiotropic cytokine that orchestrates the antitumor immune response of Th1 cells, which are primarily derived from activated APCs such as DCs and hematopoietic phagocytes (96). IL-12 acts as a heterodimeric protein of two covalently linked p35 and p40 subunits, whereas IL-12 receptor is expressed in all types of immune cells such as NK cells, T cells, and B lymphocytes (97, 98). IL-12R-2 bound ligands sequentially phosphorylate on tyrosine providing sites for the two kinases, JAK2 and TYK2 (99). Recent studies (80) have demonstrated that IL-12 blocking antibody application and IL-12-KO mice successfully offset the heightened response of ACT, therefore verifying that the effects of the gut microbiome on adoptive transfer therapy depend on IL-12. Therefore, increase in IL-12 levels might enhance the efficacy of immunotherapy by increasing the production of IFN-ɤ to stimulate the development and enhance the cytotoxicity of activated NK cells (100) and T cells and to accelerate the differentiation of CD4^+^ Th0 cells toward the Th1 phenotype, thus improving antibody-dependent cellular cytotoxicity (101).

In antibiotic-treated or GF mice, the ability of TILs to induce inflammatory cytokines, including TNF and IL-12, in response to CpG-ODN is impaired. Conversely, oral gavage with LPS has been observed to partially ameliorate the weak response (89). As a multifunctional cytotoxic molecule (102), the combination of TNF-α and its receptors (TNFR-1/TNFR-2) activates multiple signal transduction pathways, leading to diverse functions such as induction of tumor cell death by apoptosis and necrosis, stimulation of the secretion of other cytokines, and activation or recruitment of immune cells to the infection site.

All these cytokines display a synergistic trend in their biological actions. The gut bacteria that significantly prolong PFS show a preserved cytokine response to PD-1-target treatment, whereas antibiotics have been demonstrated to decrease the level of cytokines in TME (67). Therefore, the production of cytokines may be one of the mechanisms used by the gut microbiome to alter resistance to therapy.

### Promoting the Activation of DCs

Recent research in animal models and human patients with disseminated cancer has shown that the activation of tolerogenic macrophages and DCs is under the control of gut microbiome during immunotherapy. MHC-II^hi^ DCs was discovered in the tumors of *Bifidobacterium*-treated mice (40); orally feeding *B. fragilis* induced Th1 immune response in the tumor-draining lymph nodes and catalyzed the maturation of DCs in the TME, facilitating the restoration of the clinical response to CTLA-4 blockade (67).

Immature DCs are responsible for the capture, transport, and processing of antigens, and they mature in response to inflammatory signals. Upon maturation, DCs lose their phagocytic and antigen-processing capabilities (103, 104) and upregulate chemokine receptors, allowing migration to sites of activity (105). The ability of DCs to induce T-cell responses is augmented in several ways, including increased expression of surface MHC and costimulatory molecules and upregulation of soluble factors that influence the polarization of the ensuing immune response (106). PD-1 regulates the host's immunity by adjusting the threshold of antigen response and reducing the cytotoxic activity of CD8^+^ T lymphocytes (107). A recent study showed that the expression of PD-1 on DCs significantly affects the release of IL-2 and IFN-ɤ and reduces the proliferation of antigen-specific CD8^+^ T cells (108). CTLA-4 has been reported to downregulate upon differentiation into immature DCs and considerably upregulated on mature DCs (109). An increased population of CD8α^+^ DCs in the spleens of ATB-treated mice with ACT results in enhanced ability to cross-present tumor antigens to CD8^+^ T cells and increased secretion of Th1 cytokines such as IL-12 and IFN-ɤ (80).

Short-chain fatty acids (SCFAs), such as acetate, butyrate, and propionate, produced by commensal bacteria during starch fermentation, strongly modulate gene expression in human monocyte-derived DCs, thus reducing the secretion of multiple proinflammatory chemokines and inhibiting the expression of LPS-induced cytokines with enhanced activity against inflammation (110). Propionates affect DC and macrophage biology in the bone marrow and regulate Th2 cell responses in the airway of the mouse model (111). The function of gut macrophages, the most abundant immune cell type, is modulated by n-butyrate (112). The SCFAs also control several signaling pathways in immune cells (82).

### Decreasing Peripherally Derived Tregs

Specific species in the gut microbiota are associated with the production of Tregs. Patients with non-beneficial bacteria presented higher levels of Tregs in their systemic circulation, whereas patients with long-term benefit presented a low level of Tregs at the baseline (68). Smith et al. speculated that “fit bacteria” decrease peripherally derived Tregs, which is related to enhanced efficacy of PD-1-blocking therapy. Polysaccharide A, a microbial molecule produced by *B. fragilis*, promotes the development of an inducible population of CD4^+^Foxp3^+^ Tregs, a subset of Tregs, which inhibits the regulation of the immune system (113).

Tregs are evolved in the maintenance of immunological self-tolerance by actively suppressing immune lymphocytes (114). Tregs that express Foxp3, a transcription factor, are critical for the inhibition of cytokine secretion and cell contact. In a recent study, Tregs isolated from GF mice and stimulated *in vitro* with propionate showed a significant increase in Foxp3 expression, which suggested that the SCFAs could specifically induce Tregs (82). It has been reported that an increase in the number of Tregs after butyrate treatment is due to peripheral differentiation. In addition to butyrate, new-onset Treg generation in the periphery was enhanced by propionate (115).

The PD-1/PD-L1 blockade pathway increases T-cell response via Treg differentiation (116). It is presumed that beneficial bacteria and their metabolites promote the differentiation and proliferation of Tregs, leading to elevated CTLA-4 levels and improved sensitivity to CTLA-4 blockade by abrogating immunosuppression in TME (68). The SCFAs modulate the accumulation and function of the Treg pool in mice to promote the immunotherapy response.

However, opinions regarding this are not consistent. In their study on PDAC microbiome, Pushalkar et al. (59) found that, although endogenous bacterial dysbiosis triggered immune suppression, Treg differentiation did not differ in PDAC-bearing and WT mice. Similar results were observed in another study (117), where the number of Foxp3^+^ T cells isolated from the spleen was not different between PBS- and *Bifidobacterium*-treated mice, but genes targeted to certain metabolic pathways of Tregs, such as cellular macromolecules and organic substances, were expressed differentially. It remains to be verified whether the involvement of Tregs is the main cause of differential response to immunotherapy.

### Inducing the Overexpression of Chemokines

Chemokines are a family of small cytokines or signaling proteins released by cells. Some chemokines can be induced during an immune response to recruit cells of the immune system to a tumor site, and their G protein-coupled receptors are found on the surface of leukocytes. Cremonesi et al. (117) found that exposing colorectal cancer (CRC) cells to *Fusobacterium nucleatum, B. fragilis*, and *E. coli* could upregulate the majority of chemokine genes both *in vitro* and *in vivo*. They evaluated the chemokine expression levels in tumor-bearing NSG mice by intraperitoneal (i.p.) or intracecal (i.c.) injection of human CRC cells. The i.c. models showed a striking 70-fold higher level of CCL5, 19-fold higher level of CCL20, and 12-fold higher level of CXCL10 levels than those in i.p. xenografts, suggesting that exposure to gut flora resulted in a strong induction of chemokine expression. In contrast, antibiotic use significantly reduced tumor-derived chemokine expression in i.c. xenografts. Moreover, the enhanced chemokine production correlated with the gut microbiome-derived microbial load. The migration of TILs into i.c. tumors enhanced the secretion of chemokines into the TME. To summarize, gut microbiota may play a role in enhancing T-cell recruitment into the tumor site, thereby promoting the cytotoxic effect of effector immune cells toward target tumor cells to improve the clinical treatment outcome in patients. An analysis of gut microbiota constituents in CRC samples to elucidate the relationship with specific bacteria and T-cell infiltration showed that *Alloprevotella, Treponema*, and *Desulfovibrio* were abundant in tumors infiltrated with CD3^+^ T cells, whereas the overexpression of *Prevotella, Bacteroides*, and *Fretibacterium* was more frequent in CD3^low^ tumors.

In conclusion, in view of the metabolic function elicited by gut microbiota, we infer that their metabolic byproducts are perceived by cells and molecules of the immune system and modulate the equilibrium between pro- and antitumor functions. Some gut bacteria act as suppressors of inhibitory signal pathways, facilitate antigen presentation, and improve specific antitumor immunity (118). Because of the modulation of the immune system by gut microbiota, immune therapy could be tailored to focus on rejuvenating host immunity.

## Manipulation The Roles Of Gut Microbiome In Immunotherapy

The quote of Hippocrates that “all diseases begin in the gut” is consistent with the ancient theory of traditional Chinese medicine, that is, “Disease enters through the mouth.” The functions of gut microbiota are not completely known in the modulation of the immune system, metabolic equilibrium, and tumor progression. With a gradual increase in knowledge in this area, our purpose is to transit the hypothesis to practice and apply the novel therapeutic weapon in the war against cancer or other refractory diseases.

### Prediction of the Response Rate

Because of inaccuracies in predicting the response to immunotherapy, it is difficult to select patients for optimistic regimens in the clinical practice. Extensive research revealed the synergistic activity of bacteria genera including *A. muciniphila, Alistipes indistinctus, Bacteroides, Bifidobacterium, Burkholderia cepacia, Collinsella aerofaciens*, and *Enterococcus*, as well as *Faecalibacterium* and *Gemmiger formicilis* in immunotherapy. However, *Blautia obeum, Roseburia intestinalis*, and some combination of antibiotics (56, 59, 77) compromised the efficacy of immunotherapy (Table 1). It is essential to develop a candidate predictive biomarker from these aggregate data. Uniform results on the composition of the microbiota are lacking, suggesting that there may be other unidentified factors underlying this complicated process. Perhaps, a combination of commensal microbiota structure, tumor genomics, germline genetics, and other elements in a multiparameter model may likely predict clinical response to immunotherapies (55). For instance, some researchers calculated the proportion of “beneficial” and “non-beneficial” operational taxonomic units and their ratio for every patient and reached a limit conclusion—a ratio of over 1.5 indicated clinical benefit (55). Previous results have tried to utilize the gut microbiota as a potential biomarker for pancreatic cancer (123). Although the results showed a distinct response to immunotherapy in patients, it remains to be verified whether the effects will be reproduced in clinical trials.

**Table 1:** Recent studies about the regulation of immunotherapy response or related toxicity by targeting gut microbiota.

**References**	**Research object**	**Immunotherapy applied**	**Intervention and its effects on immune response or related toxicity**	**Possible mechanism**
Frankel et al. (119)	metastatic melanoma patients	ICI (including ipilimumab, nivolumab, ipilimumab plus nivolumab, and pembrolizumab)	*Bacteroides caccae*: enriched in all ICI responders	High levels of anacardic acid, as microbial metabolites, could stimulate neutrophils and macrophages, and enhance T-cell recruitment to tumor metastases.
			*Faecalibacterium prausnitzii, Bacteroides thetaiotamicron, and Holdemania filiformis*: enriched in ipilimumab plus nivolumab responders	
			*Dorea formicogenerans*: enriched in pembrolizumab responders	
Vetizou et al. (67)	metastatic melanoma patients; mice model with sarcomas	CTLA-4 blockade	*B. thetaiotaomicron or B. fragilis*: associated with the therapy efficacy	Affect IL-12-dependent TH1 immune responses.
Chaput et al. (68)	metastatic melanoma patients	CTLA-4 blockade (ipilimumab)	*Faecalibacterium genus and other Firmicutes*: longer PFS and OS;	Faecalibacterium benefit were related to lower percentage of circulating α4^+^β7^+^ T cells and CD4+ Tregs.
			*Firmicutes related phylotype:* colitis-associated;	
			*Bacteroidetes:* poor anti-cancer response, colitis-free.	
Dubin et al. (81)	metastatic melanoma patients	CTLA-4 blockade (ipilimumab)	*Bacteroidetes phylum*: resistance to the development of ICI-induced colitis.	Decreased polyamine transport and B vitamin biosynthesis were associated with an increased risk of colitis.
Sivan et al. (54)	mice model with melanoma	PD-L1 blockade	*Bifidobacterium*: nearly abolished tumor outgrowth	Augmented DC function, enhanced CD8^+^ T cell priming and accumulation in the tumor microenvironment.
Routy et al. (56)	GF or ATB-treated mice; patients with advanced cancer	PD-1 blockade	*Akkermansia muciniphila*: clinical response	Increasing the recruitment of CCR9^+^CXCR3^+^CD4^+^ T lymphocvtes into tumor beds.
Gopalakrishnan et al. (57)	melanoma patients	PD-1 blockade	*Clostridiales/Ruminococcaceae bacteria*: abundance in response patients;	High abundance of *Faecalibacterium* in the gut microbiome had an increased antigen presentation, and improved effector T cell function in the periphery or TME.
			*Bacteroidales*: enriched in non-responders;	
			Higher diversity in the fecal microbiome: significantly prolonged PFS	
Zheng et al. (120)	Patients with hepatocellular carcinoma (HCC).	PD-1 blockade	Responder-enriched species: *Akkermansia muciniphila and Ruminococcaceae spp*;	Not mentioned.
			Non-responders-increased species: *Proteobacteria*;	
Jin et al. (58)	Advanced NSCLC patients	PD-1 blockade (nivolumab)	Higher diversity of gut microbiome: prolonged PFS;	Responders had a greater frequency of unique memory CD8 T cell and NK cell subsets.
			*Alistipes putredinis, Bifidobacterium longum*, and *Prevotella copri*: abundance in responders;	
			*Ruminococcus_unclassified*: enriched in non-responding patients	
Derosa et al. (121)	advanced RCC and NSCLC patients	PD-L1 blockade	ATB treatment: decreased PFS and OS	Not mentioned.
Tanoue et al. (88)	CRC mice model	ICIs	GF mice with *11-mix*: suppressed tumor growth.	*11-mix* induced an increase in the frequency of IFNɤ^+^ CD8 TILs.
Cremonesi et al. (117)	CRC mice model	ACT	*Fusobacterium nucleatum, Bacteroides fragilis*, and *Escherichia coli* : improved survival.	Abundance of microbiota were correlated with high chemokine expression and enhanced T cell infiltration.
Uribe-Herranz et al. (80)	HPV E6/7-expressing cervical cancer mice model	ACT	Vancomycin treatment: increased ACT efficacy; Neomycin and metronidazole: unchanged.	Vancomycin-treated mice had increased systemic CD8α^+^ DCs and IL-12p70 levels and more effective expansion ACT cells.
Iida et al. (122)	antibiotics-treated or GF mice	CpG-oligonucleotide	*A. shahii*: reconstituted the TNF-producing ability; *L. fermentum*: attenuated the immune response	Antibiotic treatment induced lower cytokine production (TNF), diminished expression of pro-inflammatory gene (*Il1a, Il1b, Il12b*, and *Cxcl10)*.

### Correct Dysbiosis and Maintenance of Harmony in the Microenvironment

Based on previous studies, we expect to develop a remedial or precautionary strategy for the discordance in relation to dysbiosis of the gut microbiota in the future. The most optimal management of the commensal microbial bacteria involves FMT from healthy donors to recipients (with an illness) to correct the dysbiosis allowing colonization by the pathobionts (124). FMT is a promising method to improve the microorganism composition in the gut and is currently in use in the clinic as an optimal treatment for recurrent *Clostridium difficile* infection (125). It also has the potential to manipulate gut microbiota by FMT in patients with IBD (126, 127).

Several researchers successfully performed FMT from R patients into GF or SPF mice. The results showed that the gut microbiota of recipient mice were regrouped, and higher abundance of fit bacteria leads to a significant enrichment of innate effector cells and delayed tumor growth, compared with those in NR-FMT mice. Transplantation of sophisticated microbial populations to recipients likely leads to mucosal immune responses, either promoting or limiting the inflammation, as determined by the microbiota constituents and the genome analysis of recipients. To recapitulate, FMT could be used as a potential strategy to ameliorate the resistance to immunotherapy and enhance the therapeutic effect of those drugs.

Fecal material is complex and unpredictable, and it constantly changes the effect of FMT on the recipient's immune system. Ongoing investigations of the commensal microbiota and their functions in the host would prompt the development of probiotic agents, diet modification, and effective metabolic products derived from prebiotics or beneficial microbiota, which might eventually take the place of FMT (Table 2) (128).

**Table 2:** The promising strategies to reverse the immunotherapy resistance by manipulating gut microbiota.

**Intervention**	**Advantages**	**Disadvantages**	**Prospect**
FMT from responders	Effective in previous trials; ameliorate other immunotherapy- related symptoms	Unidentified composition and pathogenicity; Controversial safety	Select and limit transplanted organisms from a healthy donor
Prebiotic supplement	Abundance of supposedly beneficial bacteria	Display inter-individual variation; short preservation of the newly migrated microorganisms	Use patient-specific metadata and artificial intelligence to personalize dietary interventions
Microbiome-based metabolite therapy	Promising results in preliminary SCFA or flavonoids using	Unexpected interactions between metabolites and members of the microbiome to produce inactive or toxic form; Complicated chemical structure; difficult to replicate bioactive volatile metabolites under industrial settings	Require reproducible, stable and easy administered production; More potential therapeutic compounds need to be recognized
Metagenome sequencing as a tool to predict immunotherapy response	Avail in stratify responders from non-responders; Avoid resource-waste;	Complicated analysis process; Lack of consistent results	Need more reasonable standards
Proper oral antibiotics to deplete the unfavorable bacterial taxa	Apply easily and conveniently; Effective in preclinical trials	Misuse and overuse lead to dysbiosis	Specific and accurate targeting to an individual species of bacteria

For instance, some patients may have an abundance of “unfavorable” bacteria that suppress immune reaction, possibly by increasing FoxP3^+^ Tregs. Furthermore, it is posited that some antibiotics may eliminate or inhibit the growth of such bad bacteria, perhaps allowing the “good” bacteria to bloom and potentiate immune microenvironment. However, the above hypothesis has been incompatible with some studies (56), in which the application of antibiotics in patients or gnotobiotic mouse models resulted in shorter PFS and OS. From the data of existing research, it was suggested that antibiotics may play a detrimental role on clinical outcomes with ICIs. However, it is possible that inappropriate ATB use incorrectly targets “favorable” genre of intestinal microorganism and encourages bad bacteria growth. On the contrary, if bad or good bacteria are defined unequivocally and appropriate antibiotics are classified to target respective genre of bacteria, the combination use of immunotherapy and suitable antibiotics may improve the clinical response and reverse the resistance to ICBs (129).

Considering that most ATBs are highly effective and broad spectrum, they would trigger significant changes to the microenvironment due to a lack of precision to regulate specific bacterial populations. Another intervention to regulate the commensal entities include dietary changes, by which more specific essential nutrients are supplied to boost the expansion of beneficial bacteria, or conversely, unfavorable bacteria were starved to death due to scarce expected nutrients. Alternatively, immune-potentiating bacteria could be prepared as a probiotic and provided as an immunotherapy adjuvant. Instead of increasing good bacteria, selective depletion of harmful species from the community by the function of bacteriophages while maintaining the microbiota equilibrium intact could be further studied to perfect the composition of intestinal microbiota.

## Future Prospect

Although a series of investigations have verified the impact of gut microbiota on cancer immunotherapy, there still lies a great deal of ambiguity and insufficiency in these studies. The main challenge encountered is the incomplete understanding of the special microbial species involved in better immune response. Studies have proposed different types of bacteria as favorable or unfavorable in the response to immunotherapy, and a consensus has not been reached despite the studies being conducted in similar patient cohorts with the same treatments. One reason for the difference in results is that TME is unique for the type of cancer and is exclusively sensitive to the specific commensal microbiota. In addition, many studies have used FMT in experimental animals, and whether the cross-species microbiota transplantation reflects conditions in humans remains to be determined. Therefore, we anticipate that clinical syngraft trials will solve this problem.

Currently, the activity of the immune system is of interest in cancer diagnosis and treatment. Several immunotherapy protocols that involve acting on diverse molecules or pathways have emerged to dominate the current clinical therapeutic procedures. The immunological condition of the host and biology of the malignancy and tumor invasion status determine the host's response to individualized therapy. In future, we expect the use of the commensal microbiota to permeate almost every facet of clinical medical procedures. Analysis of the gut microbiome composition could potentially become a routine test for accurate evaluation of the health of cancer patients and help predict the response and adverse effects of cancer therapy. Diagnosis of dysbiosis and compensation of dysbiosis by appropriate immunogenic probiotics will increase our clinical understanding of diseases in the future.

## Author Contributions

All authors listed have made a substantial, direct and intellectual contribution to the work, and approved it for publication.

### Conflict of Interest

The authors declare that the research was conducted in the absence of any commercial or financial relationships that could be construed as a potential conflict of interest.
